# Regulatory role and mechanisms of myeloid TLR4 in anti-GBM glomerulonephritis

**DOI:** 10.1007/s00018-021-03936-1

**Published:** 2021-09-27

**Authors:** Fuye Yang, Jiaoyi Chen, Xiao Ru Huang, Wai Han Yiu, Xueqing Yu, Sydney C. W. Tang, Hui Yao Lan

**Affiliations:** 1 Department of Nephrology, The Second Affiliated Hospital of Zhejiang University School of Medicine, Hangzhou, Zhejiang 310009 People’s Republic of China; 2 Department of Medicine and Therapeutics, Li Ka Shing Institute of Health Sciences, Lui Che Woo Institute of Innovative Medicine, The Chinese University of Hong Kong, Shatin, NT, Hong Kong SAR, People’s Republic of China; 3 Guangdong-Hong Kong Joint Laboratory on Immunological and Genetic Kidney Diseases, Guangdong Academy of Medical Sciences, Guangdong Provincial People’s Hospital, Guangzhou, 510080 People’s Republic of China; 4 Division of Nephrology, Department of Medicine, The University of Hong Kong, Hong Kong SAR, People’s Republic of China; 5 The CUHK-Guangdong Provincial People’s Hospital Joint Research Laboratory on Immunological and Genetic Kidney Diseases, The Chinese University of Hong Kong, Hong Kong SAR, People’s Republic of China

**Keywords:** Myeloid TLR4, Macrophages, T cells, Anti-GBM crescentic glomerulonephritis

## Abstract

Myeloid cells and TLR4 play a critical role in acute kidney injury. This study investigated the regulatory role and mechanisms of myeloid TLR4 in experimental anti-glomerular basement membrane (GBM) glomerulonephritis (GN). Anti-GBM GN was induced in *tlr4*^*flox/flox*^ and *tlr4*^*flox/flox−lysM−cre*^ mice by intravenous injection of the sheep anti-mouse GBM antibody. Compared to control mice, conditional disruption of *tlr4* from myeloid cells, largely macrophages (> 85%), suppressed glomerular crescent formation and attenuated progressive renal injury by lowering serum creatinine and 24-h urine protein excretion while improving creatinine clearance. Mechanistically, deletion of myeloid *tlr4* markedly inhibited renal infiltration of macrophages and T cells and resulted in a shift of infiltrating macrophages from F4/80^+^iNOS^+^ M1 to F4/80^+^CD206^+^ M2 phenotype and inhibited the upregulation of renal proinflammatory cytokines IL-1β and MCP-1. Importantly, deletion of myeloid *tlr4* suppressed T cell-mediated immune injury by shifting Th1 (CD4^+^IFNγ^+^) and Th17 (CD4^+^IL-17a^+^) to Treg (CD4^+^CD25^+^FoxP3^+^) immune responses. Transcriptome analysis also revealed that disrupted myeloid TLR4 largely downregulated genes involving immune and cytokine-related pathways. Thus, myeloid TLR4 plays a pivotal role in anti-GBM GN by immunological switching from M1 to M2 and from Th1/Th17 to Treg and targeting myeloid TLR4 may be a novel therapeutic strategy for immune-mediated kidney diseases.

**Supplementary Information:**

The online version contains supplementary material available at 10.1007/s00018-021-03936-1.

## Introduction

Anti-glomerular basement membrane glomerulonephritis (anti-GBM GN) is a severe form of kidney disease characterized by rapidly progressive renal insufficiency with widespread glomerular crescent formation. It is a stereotypic autoimmune glomerular disease with the pathogenic autoantibodies targeting the epitopes on the α3 chain of type IV collagen [1–3]. Immune cells including lymphocytes, macrophages and neutrophils, intrinsic renal cells and a complex cytokine network, as well as autoantibody deposition and complement activation are involved in the pathogenesis of anti-GBM GN [4–6]. However, the mechanisms through which the cell–cell and cell–cytokine interact and regulate the pathogenesis of anti-GBM GN remain unclear.

Macrophages are a major inflammatory cell-type infiltrating the diseased kidney and have been shown to play a vital role in the pathogenesis of experimental crescentic GN as depletion of macrophages or macrophage-producing cytokines inhibits progressive kidney diseases [6–9]. Macrophages are highly heterogeneous and are versatile players in renal inflammation and fibrosis [10, 11]. Studies from animal and human crescentic GN have shown that the predominant infiltration and activation of macrophages are associated with the disease activities and the macrophage phenotypes determine the disease progression or regression as proinflammatory macrophages with M1 phenotype promote, but regulatory macrophages with anti-inflammatory M2 phenotype protect against kidney diseases [10–18]. Single-cell RNA sequencing analysis also reveals that monocytes recruited to the kidney early after injury rapidly adopt a proinflammatory and profibrotic macrophage phenotype [19]. However, the regulatory mechanisms determining macrophage polarization and alternative activations remain poorly understood. Furthermore, whether alterations of macrophage innate immunity are capable of influencing the adaptive immune response during the pathogenesis of GN remains largely unknown.

Toll-like receptors (TLRs), recognizing the exogenous and endogenous molecular patterns, have been shown to play pivotal roles in the pathogenesis of murine autoimmune GN including anti-GBM GN by modulating both the innate and adaptive immune responses [20–27]. TLR4 has been shown to contribute to the early and transient glomerular neutrophil influx at the first 24 h in nephrotoxic antibody-induced GN [20]. However, role of TLR4 in macrophage-mediated anti-GBM GN remains unclear.

To test the hypothesis that macrophages may act via TLR4 to trigger and modulate anti-GBM GN and to uncover the mechanisms through which TLR4 specifically regulates macrophage-mediated renal injury, we generated the myeloid cell-specific *tlr4* conditional knockout mice and established the anti-GBM GN model as previously described [28, 29]. Although macrophages and neutrophils are the majority of myeloid cells, neutrophil influx into the diseased kidney of anti-GBM GN is only the early and transient event [20, 30]. In contrast, the infiltration of macrophages is persistent and prominent throughout the disease course, particularly during crescentic formation. Thus, by using this mouse model, the role and mechanisms of myeloid TLR4 in the pathogenesis of anti-GBM GN were investigated.

## Materials and methods

### Mouse model of experimental anti-GBM GN

C57BL/6 mice bearing homozygous *loxP*-flanked *tlr4* (*tlr4*^*f/f*^) (JAX stock number: 024872) and *lysozyme M* promoter-driven *cre* (*lysM-cre*) (JAX stock number: 004781) were obtained from the Jackson laboratory. Mice with myeloid *tlr4* deletion (*tlr4*^*f/f−lysM−cre*^) were generated by crossing the *tlr4*^*f/f*^ mice to the *lysM-cre* mice. The genotypes of littermates were confirmed by PCR with specific primers as described by the Jackson Laboratory. All animals were raised under a specific pathogen-free condition at 25 °C with a normal 12-h light and 12-h dark cycle. Mice were allowed free access to standard food and sterilized water supplied by our animal unit.

Anti-GBM GN model was induced in the age (8–12 weeks) and gender (male and female)-matched littermates of *tlr4*^*f/f*^ and *tlr4*^*f/f−lysM−cre*^ mice according to an established protocol [28, 29]. Briefly, mice were sensitized by subcutaneous injection of 2 mg of sheep globulin in 200 µL of Freund’s complete adjuvant (Sigma Aldrich, St. Louis, Missouri, USA) in each flank. Anti-GBM GN was initiated by intravenous administration of 5 mg of sheep anti-mouse GBM globulin via tail vein 10 days later. Groups of age- and gender-matched *tlr4*^*f/f*^ and *tlr4*^*f/f−lysM−cre*^ mice without disease induction were used as controls. All the experimental procedures were approved by the Animal Experimentation Ethnics Committee of the Chinese University of Hong Kong and in accordance with the relevant guidelines and regulations.

### Measurement of proteinuria and creatinine

Urine samples were collected in metabolic cages before and after induction of disease on days 0, 1, 3, 7and 14. The 24 h urinary protein and the urinary microalbumin were analyzed according to the manufacturer's instructions as previously described [28, 29]. Urinary albumin excretion was expressed as total urinary albumin/creatinine (in micrograms per milligram). The creatinine clearance was calculated using the following formula: [urine creatinine (mg/dl) × 24 h urine volume (ml)]/[24 × 60(min) × serum creatinine (mg/dl)].

### Flow cytometry analysis

Kidney single cell suspensions for flow cytometry were prepared as previously described [29]. Single-cell suspensions were then incubated with Fc blocker (BD Biosciences, San Jose, California) for 30 min on ice with 10% FBS in PBS and then stained with pre-conjugated antibody cocktails in dark for 30 min on ice. For the intracellular staining, cell suspensions were fixed with IC Fixation Buffer and permeabilized with Permeabilization Buffer (eBioscience). Cells without or with irrelevant antibody staining were used as negative controls. DAPI was used to distinguish the live and dead cells. The antibodies used in the study were as follows: CD45-FITC, -PE (eBioscience, clone: 30-F11); CD11b-Alex 488, -PE (eBioscience, clone: M1/70); F4/80-FITC, -PE, -APC, Pacific blue (eBioscience, clone: BM8); Ly6G-FITC (eBioscience, clone: 1A8); iNOS-APC (eBioscience, clone: CXNFT); CD206 (Biolegend, clone: C068C2); MHC II (I-A/I-E)-APC (eBioscience, clone: M5/114.15.2); CD3-FITC, -PE (BD, clone: 17A2); CD4-FITC, -PE, -APC (eBioscience, clone: GK1.5); IFNγ (eBioscience, clone: XMG1.2); IL-4-APC (eBioscience, clone: 11B11); IL-17a-APC (eBioscience, clone: eBio 1787); CD25-PE (eBioscience, clone: PC61.5); Foxp3-APC (eBioscience, clone: FJK-16s); Ly6C-PE (eBioscience, clone: HK1.4). Flow cytometry was performed on a BD LSRFortessaTM using the FlowJo software v10. The percent changes in the number of different subpopulations infiltrating the kidney with anti-GBM GN were quantitatively analyzed against the total kidney cell counts isolated from the entire left mouse kidney.

### Histology and immunohistochemistry

Methyl Carnoy’s fixed, paraffin-embedded kidney sections (4 μm) were deparaffinized and stained with periodic acid Schiff (PAS). Segmental glomerular necrosis and crescent formation were scored by counting at least 50 glomeruli on PAS-stained section of each mouse and expressed as the percentage of total glomeruli examined. Immunohistochemistry was performed in paraffin sections with monoclonal antibodies to neutrophils (NIMP-R14) (Santa Cruz Biotechnology, Santa Cruz, CA), macrophages (F4/80) (Serotec, Oxford, UK) and a rabbit polyclonal antibody to CD3^+^ T cells (SP7) (Abcam, Cambridge, UK). The number of positive cells for NIMP-R14 and F4/80 was counted in 20 consecutive glomeruli and expressed as cells per glomerular cross-section (gcs), while positive cells in the tubulointerstitium were counted under high-power fields (400 × magnification) by means of a 0.0625 mm^2^ graticule fitted in the eyepiece of the microscope and expressed as cells per mm^2^.

### Immunofluorescence

CD4 cells infiltrating in the kidney were identified in snap-frozen sections (4 μm) by immunofluorescence with Dylight 550-rat anti-mouse CD4 monoclonal antibody (Leinco Technologies, St. Louis, Missouri, USA). Sections were counterstained with DAPI and examined under a Zeiss Axioplan2 imaging microscope (Carl Zeiss, Oberkoche, Germany).

### ELISA

Levels of MCP-1 in kidney homogenates were measured using ELISA kits (R&D Systems, Minneapolis, USA) following the manufacturer’s instructions as described previously. Briefly, the snapped frozen renal cortical tissue was homogenized with ultrasonication in PBS on ice. Then, the samples were centrifuged at 12,000*g* at 4 °C for 10 min, the supernatant was collected and total protein was quantified followed by ELISA analysis. The final levels of interested cytokines were normalized to pg per mg of renal cortex tissue.

### Real-time PCR

Total mRNAs were isolated using the RNeasy Kit according to the manufacturer’s instructions (Qiagen, Düsseldorf, Germany). The cDNA was synthesized, and real-time PCR was performed on an Opticon 2 real-time PCR machine (Bio-Rad Laboratories, Hercules, California, USA) using the IQ SYBR Green supermix reagent (Bio-Rad Laboratories) as described previously [28, 29]. The primers used in this study, including mouse MCP-1, IL-1β, IFN-γ, IL-17a and GAPDH, were as mentioned previously [28, 29]. The ratio of interested mRNA was normalized to GAPDH mRNA expression.

### PolyA RNA sequencing and functional enrichment analysis

Bone marrow-derived macrophages (BMDMs) isolated from *tlr4*^*f/f*^ and *tlr4*^*f/f−lysM−cre*^ mice were stimulated with or without 1 µg/mL of LPS for 6 h. Three samples of total mRNAs from each group were subjected for polyA RNA sequencing. The libraries were paired end sequenced (PE150, Sequencing reads were 150 bp) at Guangzhou RiboBio Co., Ltd. (Guangzhou, China) using Illumina HiSeq3000 platform. The clean reads were obtained after removal of reads containing adapter, ploy-N and at low quality from raw data. HISAT2 was used to align the clean reads to the mouse reference genome mm10 with default parameters. HTSeq was employed to convert aligned short reads into read counts for each gene model. Differential gene expression was assessed by DESeq using read counts as input. Differentially expressed genes (DEGs) were chosen according to the criteria of fold change > 2 and false discovery rate (FDR) adjusted *p*-value < 0.01. Network enrichment analysis was built under Metascape (http://metascape.org/) [31]. The sequencing data are available at the Gene Expression Omnibus website (https://www.ncbi.nlm.nih.gov/geo/) under accession GSE162497. To review GEO accession GSE162497, go to https://www.ncbi.nlm.nih.gov/geo/query/acc.cgi?acc=GSE162497 and enter the secure reviewer token into to box: cvstamiybjojtuz.

### Statistics

All of the statistical tests were performed using Prism 5.0 GraphPad Software (GraphPad Software, La Jolla, California, USA). Data obtained from this study were expressed as the mean ± SEM. Two-group comparisons were performed using an independent sample *t*-test unless otherwise indicated. Multiple group comparisons were performed using one-way analysis of variance (ANOVA) followed by Tukey’s post hoc tests. Differences with a *p* value less than 0.05 were considered statistically significant.

## Results

### Deficiency of myeloid TLR4 ameliorates experimental anti-GBM GN

**Fig. 1:**
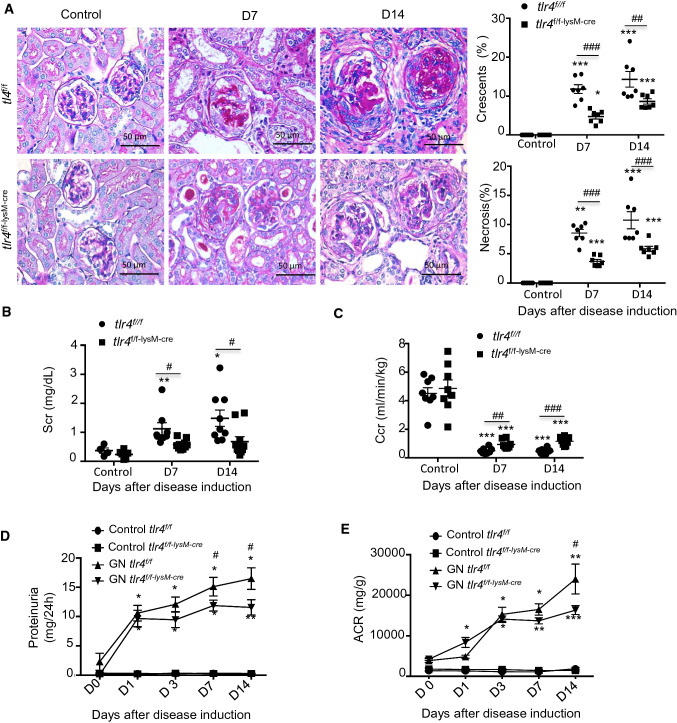
Deficiency of myeloid TLR4 ameliorates experimental anti-GBM GN. **A** Representative images and quantification of glomeruli crescents and segmental necrosis in PAS sections (magnification × 100). The quantification is expressed as the percentage of glomeruli with crescent or segmental sclerosis, mean ± SEM (ANOVA, *n* = 3–8). **B** Serum creatinine (Scr), mean ± SEM (ANOVA, *n* = 4–10). **C** Creatinine clearance rate (Ccr), mean ± SEM (*t *test, *n* = 8). **D** 24-h urine protein, mean ± SEM (ANOVA, *n* = 7–10). **E** Urine albumin creatinine ration (ACR) over the disease course, mean ± SEM (ANOVA, *n* = 7–10). Each dot represents one mouse. *p < 0.05, **p < 0.01, ***p < 0.001 versus corresponding control; #*p* < 0.05, ##*p* < 0.01, ###*p* < 0.001 versus corresponding *tlr4*^*f/f*^. Scale bar, 50 μM

The conditional myeloid *tlr4* knockout mice were generated by crossing *tlr4*^*f/f*^ mice to *lysM-cre* mice (S. Figure 1A). Deletion was then assessed both in BMDMs isolated from *tlr4*^*f/f*^ and *tlr4*^*f/f−lysM−cre*^ mice (S. Figure 1B and C) and in the whole kidneys after induction of experimental anti-GBM GN by real-time PCR analysis (S. Figure 1D) or flow cytometry (S. Figure 1E).

To determine the functional significance of myeloid TLR4 in anti-GBM GN, both *tlr4*^*f/f*^ and *tlr4*^*f/f−lysM−cre*^ mice were subjected to the induction of anti-GBM GN. Data showed that 7 and 14 days after administration of anti-GBM antibody, the *tlr4*^*f/f*^ mice developed severe renal injuries including segmental glomerular capillary necrosis and glomerular crescent formation (Fig. 1A), elevated serum creatinine and a fall in creatinine clearance (Fig. 1B, C) and an increase in 24-h urinary protein excretion and the urine albumin/creatine ratio (Fig. 1D, E). In contrast, these renal pathological and functional injuries were markedly attenuated in *tlr4*^*f/f−lysM−cre*^ mice (Fig. 1A–E), demonstrating a pathogenic role of myeloid TLR4 in anti-GBM GN.

### Deficiency of myeloid TLR4 inhibits macrophage-dominant renal infiltrates in experimental anti-GBM GN

**Fig. 2:**
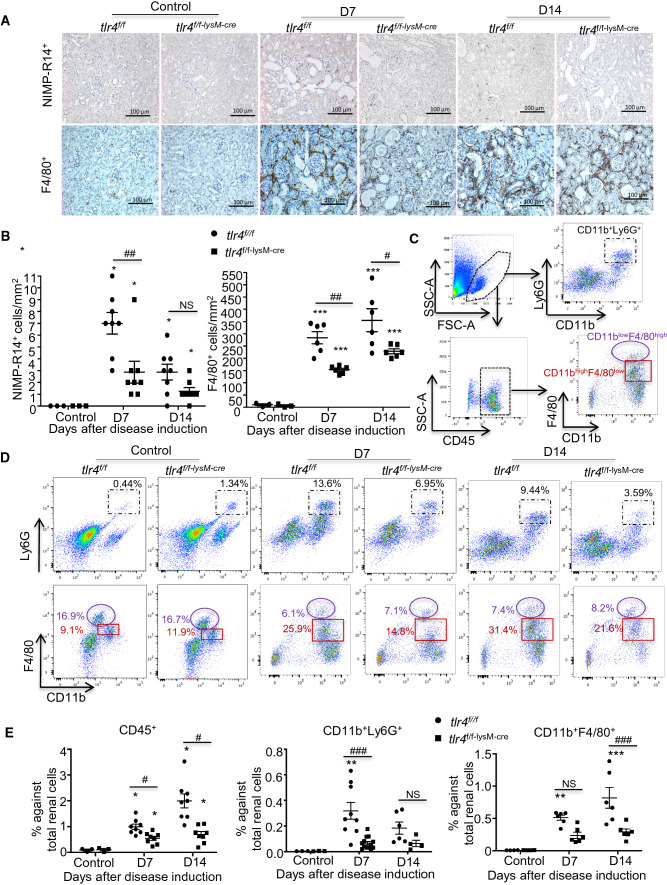
Deficiency of myeloid TLR4 inhibits macrophage-dominant renal infiltrates in experimental anti-GBM GN. **A** Representative images of neutrophil (NIMP-R14^+^) and macrophage (F4/80^+^) accumulation in kidney sections (magnification × 40). **B** Semiquantitative data of **A**, mean ± SEM (ANOVA, *n* = 3–8). **C** Gating strategy of flow cytometric analysis of renal singlets. **D** Representative flow cytometry plots of renal singlets from *tlr4*^*f/f*^ and *tlr4*^*f/f−lysM−cre*^ mice of control condition or after induction of anti-GBM GN at indicated time-point for CD45^+^ leukocytes, CD11b^+^Ly6G^+^ neutrophils and CD11b^+^F4/80^+^ macrophages (CD11b^low^F4/80^high^ and CD11b^high^F4/80^low^). **E** Statistical data of C and D, mean ± SEM (ANOVA, *n* = 3–8). Each dot represents one mouse. *p < 0.05, **p < 0.01, ***p < 0.001 versus corresponding control; #*p* < 0.05, ##*p* < 0.01 versus corresponding *tlr4*^*f/f*^. Scale bar, 100 μm

To explore the mechanisms whereby deletion of myeloid TLR4 inhibits anti-GBM GN, we first examined the innate immune responses by immunohistochemical detection of neutrophils and macrophages infiltrating the diseased kidney. Consistent with previous notion that neutrophils are not persistently involved in the progression of anti-GBM GN as the influx is peaking at 2–3 h and is resolving within the first 24 h during the disease course [20, 30], we found only a few NMP-R14^+^ neutrophils detectable in the diseased kidneys of *tlr4*^*f/f*^ and *tlr4*^*f/f−lysM−cre*^ mice at day 7 and 14 after anti-GBM crescentic GN induction, accounting for 3–11 NMP-14^+^ cells/mm^2^ (Fig. 2A, B). In contrast, massive F4/80^+^ macrophages accumulated in the diseased kidneys of *tlr4*^*f/f*^ mice, accounting for 315 ± 27 and 395 ± 51 F4/80^+^ cells/mm^2^ at day 7 and 14, respectively, over the disease course, which was largely blunted in *tlr4*^*f/f−lysM−cre*^ mice (Fig. 2A, B). Flow cytometry also revealed that the majority of infiltrating inflammatory cells in the kidney are CD11b^+^F4/80^+^ macrophages, accounting for more than 80% of CD45^+^ cells, whereas the CD11b^+^Ly6G^+^ population was a relatively small proportion (< 20%) (Fig. 2CE), of which more than 85% of TLR4 was deleted from CD11b^+^F4/80^+^ populations when compared to CD11b^+^Ly6G^+^ cells (Fig. 2D). Interestingly, further quantitative analysis of total kidney cell counts harvested from the entire left mouse kidney showed that while deletion of myeloid *tlr4* largely suppressed CD45^+^ cells infiltrating the left kidney in both day 7 and day 14 after disease induction, it significantly suppressed CD11b^+^Ly6G^+^ cells at day 7 but CD11b^+^F4/80^+^ cells at day 14 after anti-GBM GN induction (Fig. 2E). These observations demonstrated that the majority of myeloid TLR4 was deleted from macrophages, to a less extent of neutrophils, and macrophage TLR4 may play a critical role in the pathogenesis of anti-GBM GN.

**Fig. 3:**
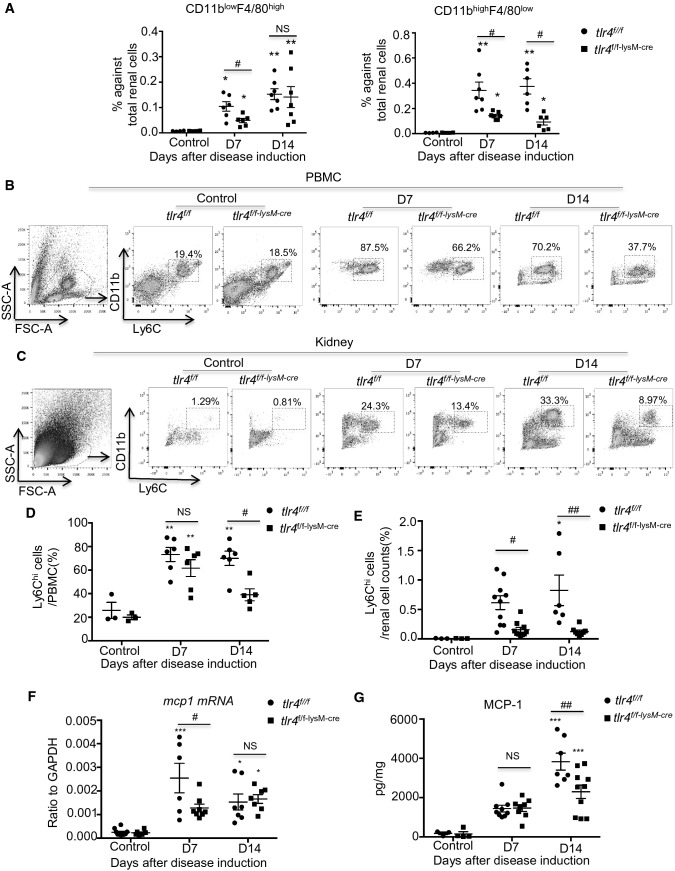
Deficiency of myeloid TLR4 suppresses peripheral expansion and renal recruitment of Ly6C^hi^ cells in experimental anti-GBM GN. **A** Subset analysis of macrophages in renal singlets by flowcytometry with embryonic (CD11b^low^F4/80^high^) and monocyte (CD11b^high^F4/80^low^) origin, mean ± SEM (ANOVA, *n* = 3–7). **B, C** Representative flow cytometry plots for CD11b^+^Ly6C^+^ cells from peripheral blood (**B**) and renal singlets (**C**). **D, E** Quantitative analysis of Ly6C^+^ cells in peripheral blood (**B**) and kidney (**E**), mean ± SEM (ANOVA, *n* = 3–10). **F** Real-time PCR analysis of renal MCP-1 mRNA expression, mean ± SEM (ANOVA, *n* = 5–8). **G** ELISA analysis of renal MCP-1 protein expression, mean ± SEM (ANOVA, *n* = 4–10). Each dot represents one mouse. *p < 0.05, **p < 0.01, ***p < 0.001 versus corresponding control; #*p* < 0.05, ##*p* < 0.01 versus corresponding *tlr4*^*f/f*^

We next investigated the role of myeloid TLR4 in the recruitment of renal inflammatory macrophages by identifying their origin from either embryonic (CD11b^low^F4/80^high^) or monocyte-derived (CD11b^high^F4/80^low^) macrophages using flow cytometry [32, 33]. Results shown in Fig. 3A revealed that deletion of myeloid *tlr4* mainly reduced the monocyte-derived CD11b^high^F4/80^low^ subset infiltrating the diseased kidney, instead of the CD11b^low^F4/80^high^ embryonic macrophages. These observations imply that myeloid TLR4 may contribute to the recruitment of monocyte-derived inflammatory macrophages into the inflamed kidney to mediate anti-GBM GN.

### Deficiency of myeloid TLR4 suppresses peripheral expansion and renal recruitment of Ly6C^+^ monocytes and promotes macrophage polarization from M1 towards M2 in experimental anti-GBM GN

Ly6C is widely used to identify the functionally discrete monocyte/macrophage subpopulations [34, 35]. We thus determined the influence of myeloid TLR4 on distinct monocyte/macrophage subsets in PBMCs and kidneys following anti-GBM GN induction by flow cytometry. As shown in Fig. 3B and D, the peripheral CD11b^+^Ly6C^hi^ population was expanded in *tlr4*^*f/f*^ mice at day 7 and 14 after anti-GBM antibody injection, resulting in an increase in the renal recruitment of CD11b^+^Ly6C^hi^ cells in *tlr4*^*f/f*^ mice (Fig. 3C, E). In contrast, this peripheral expansion and renal infiltration of CD11b^+^Ly6C^hi^ population was inhibited in *tlr4*^*f/f−lysM−cre*^ mice (Fig. 3B–E). Further studies revealed that this inhibitory effect on the renal recruitment of CD11b^+^Ly6C^hi^ in the *tlr4*^*f/f−lysM−cre*^ mouse was largely attributed to the inhibition of monocyte chemotactic protein-1 (MCP-1) as determined at the mRNA levels by realtime PCR (Fig. 3F) and at the protein levels by ELISA (Fig. 3G).

**Fig. 4:**
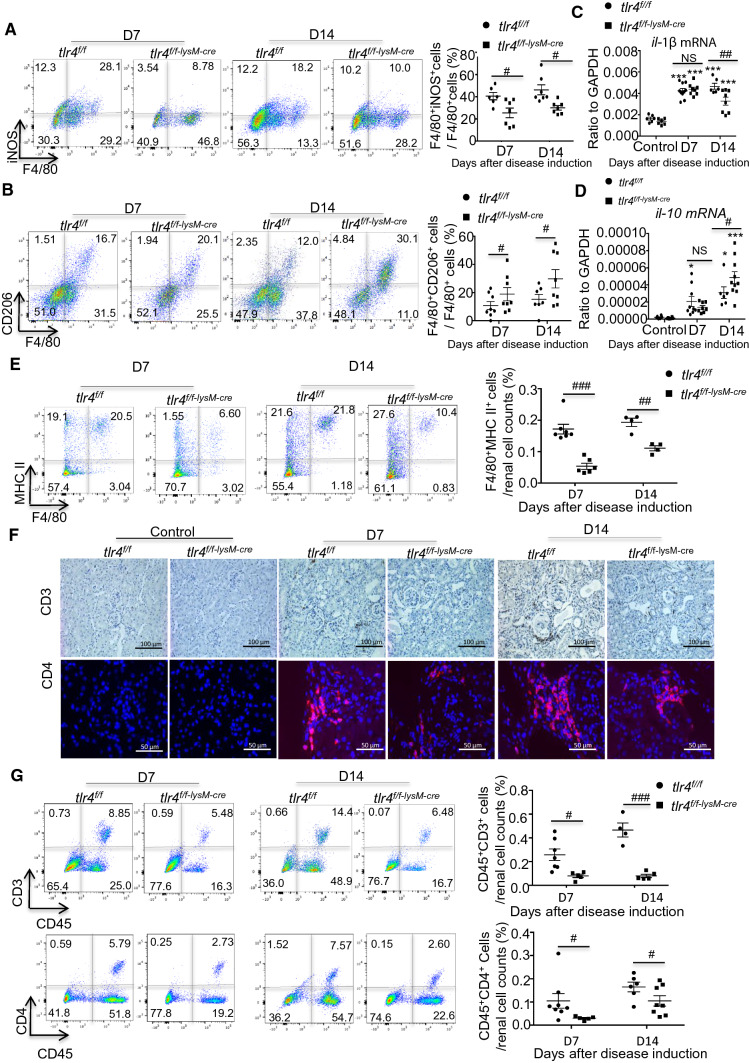
Deficiency of myeloid TLR4 promotes macrophage polarization from M1 towards M2, blunts macrophage MHC II expression and further reduces T cell accumulation in experimental anti-GBM crescentic GN. Representative flow cytometry plots and quantification of renal singlets analyzed for **A** M1 (F4/80^+^iNOS^+^), mean ± SEM (*t* test, *n* = 6–7) and **B** M2 (F4/80^+^CD206^+^), mean ± SEM (*t* test, *n* = 7–8) (both gated on CD45^+^ cells). Realtime PCR analysis of **C** *il-1β,* mean ± SEM (ANOVA, *n* = 6–10) and **D** *il-10,* mean ± SEM (ANOVA, *n* = 6–10) in kidneys isolated from control or diseased mice on day7 and 14 after anti-GBM GN induction. **E** Representative flow cytometry plots and quantification of renal singlets analyzed for F4/80^+^MHC II^+^ cells. Gated on CD45^+^ cells, mean ± SEM (*t* test, *n* = 4–6). **F** Representative kidney sections stained for CD3^+^ (magnification × 40) and CD4^+^T cells, (magnification × 100). **G** Representative flow cytometry plots and quantification of renal singlets analyzed for CD3^+^, mean ± SEM (*t *test, *n* = 4–7) and CD4^+^ T cells, mean ± SEM (*t* test, *n* = 5–8) (Gated on leukocyte in FSC vs. SSC plot). Each dot represents one mouse. *p < 0.05, ***p < 0.001 versus corresponding control; #*p* < 0.05, ##*p* < 0.01 versus corresponding *tlr4*^*f/f*^

The Ly6C^hi^ monocytes recruited into the tissue are believed to immediately differentiate into the classically activated macrophages to exert the proinflammatory and immunogenic functions in many diseases. [34, 35] In addition to the lineage differentiation, mature macrophages can adopt various phenotypes and functions depending on the environmental context. [14] Thus, the effect of myeloid TLR4 on macrophage polarization during the disease course of anti-GBM GN was further analyzed. As shown in Fig. 4A and B, the *tlr4*^*f/f*^ mice presented with an F4/80^+^iNOS^+^ M1-predominant macrophage subpopulation, whereas the *tlr4*^*f/f–lysM−cre*^ mice exhibited an F4/80^+^CD206^+^ M2-predominant subpopulation at days 7 and 14 after anti-GBM GN induction. Real-time PCR also revealed that deletion of myeloid TLR4 significantly inhibited proinflammatory cytokine *il-1β* mRNA but increased anti-inflammatory cytokine *il-10* mRNA expression at day 14 although these were not seen at day 7 after anti-GBM antibody injection (Fig. 4C, D).

### Deficiency of myeloid TLR4 blunts the immunogenic activity of macrophages and suppresses T cell-mediated experimental anti-GBM GN by shifting the Th1/Th17 towards the T reg immune responses

It is well known that activated macrophages can acquire the antigen-presenting capacity of the exogenous antigen to CD4^+^ T cells via the MHC II molecules together with the co-stimulators. Here we found that deficiency of myeloid TLR4 largely reduced the MHC II expression by macrophages infiltrating the kidney in *tlr4*^*f/f–lysM−Cre*^ mice as compared to those in *tlr4*^*f/f*^ mice at both days 7 and day 14 after anti-GBM GN induction (Fig. 4E). It is well recognized that T cells, presumably CD4^+^ T cells, play a pivotal role in anti-GBM GN. [3–5] An interesting finding in the present study was that disruption of myeloid TLR4 largely suppressed the CD3^+^ T cells and CD4^+^ Th cells infiltrating the diseased kidneys as determined by flow cytometry and immunohistochemistry (Fig. 4 F and G).

**Fig. 5:**
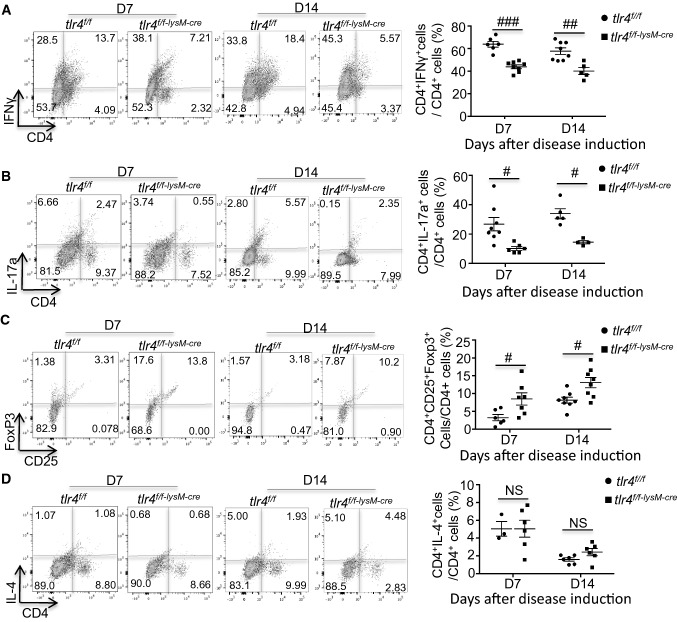
Deficiency of myeloid TLR4 suppresses T cell-mediated experimental anti-GBM crescentic GN by shifting the Th1/Th17 towards the Treg immune responses. Representative flow cytometry plots of renal singlets analyzed for: **A** Th1 (CD4^+^IFNγ^+^), mean ± SEM (*t *test, *n* = 5–8); **B** Th17 (CD4^+^IL17a^+^), mean ± SEM (*t*-test, *n* = 5–8); **C.** Treg (CD4^+^CD25^+^ Foxp3^+^), mean ± SEM (*t *test, *n* = 6–8); and **D** Th2 (CD4^+^IL-4^+^), mean ± SEM (*t *test, *n* = 3–6) (Gated on CD4^+^ cells for Treg, otherwise gated on CD45^+^). Each dot represents one mouse. #*p* < 0.05, ##*p* < 0.01, ###*p* < 0.001 versus corresponding *tlr4*^*f/f*^

It has been well established that in anti-GBM and autoimmune GN, Th1 and Th17 are two major CD4^+^ subpopulations responsible for a rapidly progressive renal injury, [36–38] while Treg subpopulation is renal protective. [28, 29] We next examined if deletion of myeloid *tlr4* influenced the immune profile of CD4^+^ T cells by examining Th1 (CD4^+^IFNγ^+^), Th2 (CD4^+^IL-4^+^), Th17 (CD4^+^IL-17a^+^) and Treg (CD4^+^CD25^+^FoxP3^+^) subpopulations. Flow cytometry demonstrated that disrupted myeloid TLR4 resulted in a significant reduction in CD4^+^IFNγ^+^ Th1 and CD4^+^IL17a^+^ Th17 cells infiltrating the diseased kidney in *tlr4*^*f/f−lysM−Cre*^ animals compared to the *tlr4*^*f/f*^ mice (Fig. 5A, B). This was accompanied by an increase in CD4^+^CD25^+^Foxp3^+^ Treg subpopulation (Fig. 5C), although there was not significant change in the CD4^+^IL4^+^ Th2 subpopulation between *tlr4*^*f/f−lysM−Cre*^ and *tlr4*^*f/f*^ mice (Fig. 5D). These data imply that myeloid TLR4 plays a regulatory role in adaptive immunity and deletion of macrophage *tlr4* can alter the T cell immunity by shifting the Th1/Th17 to Treg immune responses during the development of anti-GBM GN.

### Deficiency of myeloid TLR4 downregulates the genes involving the proinflammatory and immunogenic Pathways

**Fig. 6:**
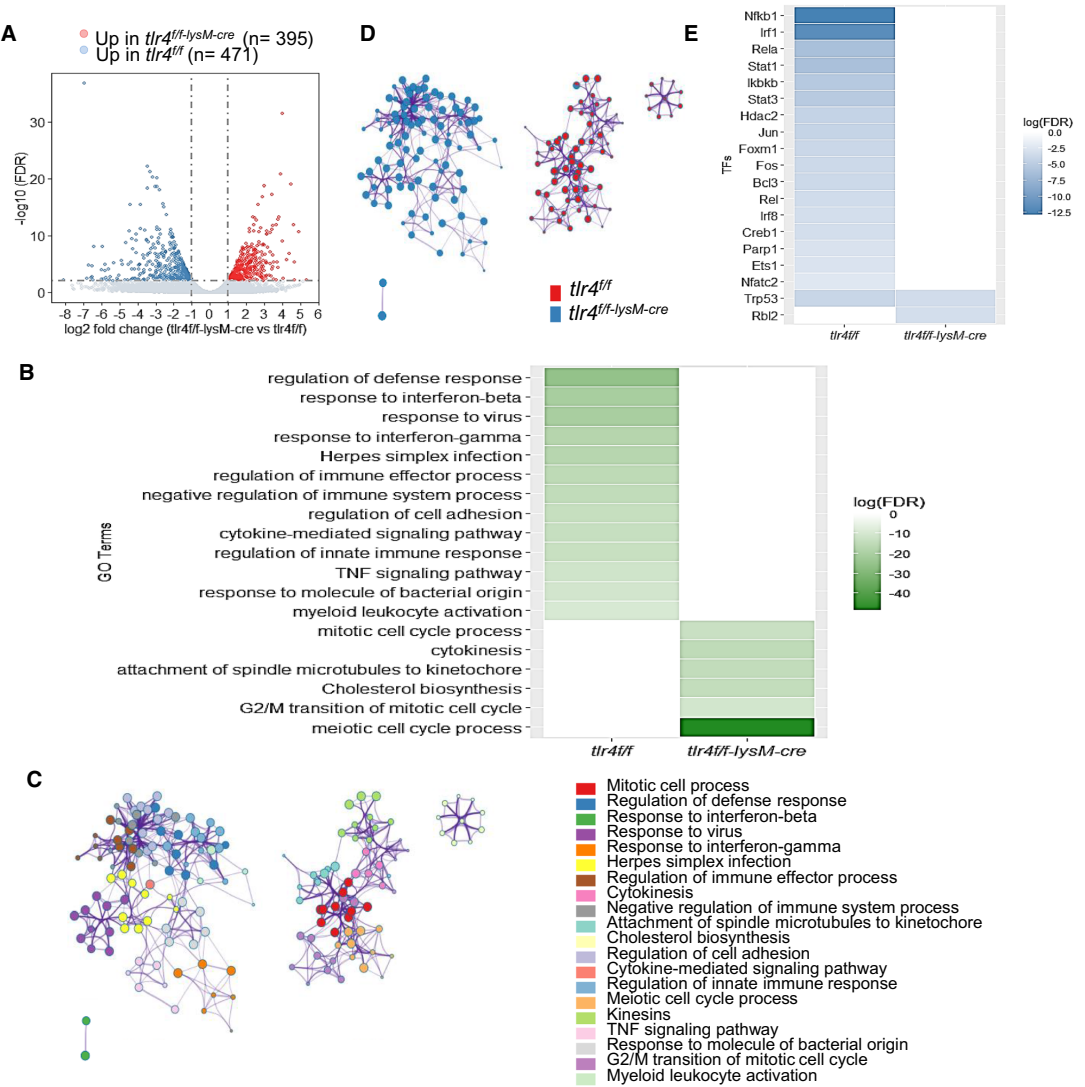
Deficiency of myeloid TLR4 alters the gene expression profile of macrophages from the proinflammatory and immunogenic state to the quiescent state. **A** The volcano plot of transcriptomic changes between LPS stimulated *tlr4*^*f/f−lysM−Cre*^ and *tlr4*^*f/f*^ BMDMs; **B** Heatmap shows top significantly enriched terms across up-regulated DEGs in LPS treated *tlr4*^*f/f*^ and *tlr4*^*f/f−lysM−Cre*^ BMDMs, coloured by log (FDR); **C** Enrichment network of 866 DEGs between LPS stimulated *tlr4*^*f/f*^ and *tlr4*^*f/f−lysM−cre*^ BMDMs. Each node represents one enriched term. The number of input genes falling into each term is represented as the circle size and cluster identities are distinguished by colours; **D** Network of enriched terms represented as pie charts, where the sector size is proportional to the number of up-regulated DEGs originated from each sample group. **E** Heatmap shows top significantly enriched transcriptional regulatory interaction of up-regulated DEGs in LPS treated *tlr4*^*f/f*^ and *tlr4*^*f/f−lysM−Cre*^ BMDMs, coloured by log (FDR). All the enrichment analysis has been carried out with the following ontology sources: KEGG Pathway, GO Biological Processes, Reactome Gene Sets, CORUM, TRRUST and PaGenBase. Terms with a *p*-value < 0.01, a minimum count of 3, and an enrichment factor > 1.5 (the enrichment factor is the ratio between the observed counts and the counts expected by chance) are collected and grouped into clusters based on their membership similarities. DEGs, differentially expressed genes; FDR, false discovery rate

To explore the downstream molecules responsible for the regulatory role of myeloid TLR4 during inflammatory responses, we employed polyA RNA sequencing to analyze transcriptome profiles of *tlr4*^*f/f*^ and *tlr4*^*f/f−lysM−Cre*^ BMDMs with or without LPS stimulation (S. Figure 2A). Individual replicates in each group were clearly clustered and highly homologous within group according to their gene expression profiles and overall expression similarity (S. Figure 2B and C). Although some genes (*n* = 957) associated with inflammatory response and cytokine-mediated signaling pathways were up-regulated (FDR < 0.01, and > twofold differences in expression) in both of the LPS-stimulated groups comparing to their unstimulated control, respectively (S. Figure 3A and B), there was still a large subset of genes whose induction by LPS was significantly suppressed by deficiency of macrophage TLR4 (S. Figure 3C). Comparing the transcriptome profiles between LPS challenged *tlr4*^*f/f*^ and *tlr4*^*f/f−lysM−cre*^ BMDMs, 866 DEGs were recognized (Fig. 6A, S. Figure 3C). Among them, 471 genes were upregulated in LPS treated *tlr4*^*f/f*^ BMDMs, most of which were highly enriched with Gene Ontology (GO) terms “regulation of defense response”, “response to interferon-beta”, “response to interferon-gamma” and “TNF signaling pathways”. Efferocytosis is the signature of macrophage activation, thus the efferocytosis-related gene expression was further analyzed by heatmap. Results shown in Supplementary Fig. 4 revealed that LPS stimulation upregulated the genes that are promoting macrophage efferocytosis, such as Abca7, C2, C3, Ccl2, Tgm2, Rhog, Jmjd6, Xkr8, Adgrb1, Thbs1, Mertk, Tyro3 and Itgav, but downregulated the genes that are suppressing macrophage efferocytosis, including Trem2, Cd300lf, Hmgb1 and Rab14 in *tlr4*^*f/f*^ BMDMs, which were reversed by deleting macrophage *tlr4* in *tlr4*^*f/f−LysM−cre*^ BMDMs. Instead, 395 significantly upregulated DEGs in LPS treated *tlr4*^*f/f−lysM−cre*^ BMDMs were mostly involved in “meiotic cell cycle process”, “G2/M transition of mitotic cell cycle” and “Cholesterol biosynthesis” as demonstrated in Fig. 6B. Enrichment network of the 866 DEGs further illustrated the heterogeneity in LPS triggered downstream biology process due to TLR4 deficiency (Fig. 6C, D). At last, TRRUST [39] database from Metascape was employed to explore the candidate downstream transcription factors (TFs) modulating the up-regulated DEGs in LPS treated *tlr4*^*f/f*^ or *tlr4*^*f/f−lysM−cre*^ BMDMs. Apart from the classic TFs (Nfkb1, Jun and Fos) in TLR4 signaling pathway, the proinflammatory M1 macrophage phenotype-driving TFs: Irf1 and Irf8 [40], together with Stat1 and Stat3 which are responsible for Th1 and Th17 cell differentiation respectively [41, 42], were also found to be the key TFs for TLR4-dependent DEGs in LPS treated *tlr4*^*f/f*^ BMDMs. Meanwhile, only Rbl2, known as a regulator of cell division, was identified as potential candidate TF having significant interactions with up-regulated DEGs in LPS treated *tlr4*^*f/f−lysM−cre*^ BMDMs (Fig. 6E).

## Discussion

Systemic *tlr4* knockout (KO) has been shown to exert renoprotective effects in number of renal diseases, including diabetic nephropathy, lupus nephritis and nephrotoxic nephritis [20–27]. However, since TLR4 are constitutively expressed by a variety of cells including the intrinsic renal cells such as tubular epithelial cells, endothelial cells and glomerular cells, and the extrinsic inflammatory cells such as neutrophils and macrophages, findings from the systemic *tlr4* KO mouse models may just suggest the overall role of TLR4 in progressive renal injury. In the present study, we developed a conditional myeloid *tlr4* KO mouse and uncovered the role and mechanisms of myeloid TLR4 in a mouse model of anti-GBM GN. We found that myeloid TLR4 was largely deleted from both peripheral and renal infiltrating macrophages to a less extent of neutrophils, resulting in protection against anti-GBM GN in terms of glomerular crescent formation, segmental necrosis, urine protein excretion and renal function. These observations revealed a critical role for myeloid TLR4, particularly macrophage TLR4, in the pathogenesis of anti-GBM GN.

It has been well recognized that macrophages play a pivotal role in progressive renal injury including glomerular crescentic formation [6–12]. The present study unraveled that upregulation of TLR4 on macrophages may trigger macrophage polarization towards M1 phenotype to actively produce proinflammatory cytokines, resulting in a progression of anti-GBM GN. This was confirmed by deleting myeloid TLR4 to suppress the M1 macrophage accumulation and activation while to promote their differentiation into the anti-inflammatory M2 phenotype to protect against the anti-GBM GN. These results suggest that myeloid TLR4 may play a critical role by modulating macrophages in the pathogenesis of anti-GBM GN. Indeed, CD11b^+^Ly6C^hi^ population correlates with the proinflammatory macrophages and carries the proinflammatory characteristics by expressing high levels of proinflammatory cytokines [34, 35]. In the present study, we found that TLR4 tightly regulated the CD11b^+^Ly6C^hi^ peripheral expansion and the renal recruitment via the MCP-1 dependent mechanism as deletion of myeloid *tlr4* inhibited renal MCP-1 expression and the peripheral and renal CD11b^+^Ly6C^hi^ population expansion, thereby largely inhibiting M1 proinflammatory macrophage while promoting M2 anti-inflammatory macrophage accumulation in the diseased kidneys of anti-GBM GN. The in vitro study further confirmed that upon LPS stimulation, deletion of TLR4 greatly suppressed genes involved in proinflammatory signaling and M1 polarization. These findings provided a direct evidence for a necessary role of TLR4 in macrophage-mediated anti-GBM GN.

The most significant finding from this study was that myeloid TLR4 also plays a modulatory role in T cell polarization and activation as deletion of myeloid *tlr4* dampened Th1/Th17 but enhanced Treg immune response, therefore protecting T cell-mediated anti-GBM GN. It is well established that the Th1 and Th17 immune responses contribute significantly to the pathogenesis of anti-GBM GN [36–38], whereas Treg cells are protective [28, 29]. The present study demonstrated that macrophages could orchestrate the T cell response rather than being directed by T cells. Injection of anti-GBM antibody induced the M1-predomiant activities with increased proinflammatory cytokines such as MCP-1 to recruit more Ly6C^hi^ monocytes to the inflamed site and to promote macrophage activation and differentiation into antigen-presenting cells with high levels of MHC class II expression, which may subsequentially present the antigen to the CD4^+^ T cells as recently reported [43]. The interaction between immunogenic macrophages and CD4^+^ T cells may direct T cell activation and production of Th1/Th17-like cytokines, such as IFNγ and IL-17, resulting in Th1/Th17-mediated anti-GBM GN. In contrast, loss of macrophage TLR4 resulted in the shift of macrophages from M1 to M2-predominant phenotype, thereby suppressing the immunogenicity of MHC class II expressing macrophages and redirecting T cell immune responses from Th1/Th17 towards Treg to protect against anti-GBM GN. Two candidate transcription factors Stat1 and Stat3 that promote Th1/Th17 differentiation were further identified and specifically enriched from up-regulated DEGs in LPS treated *tlr4*^*f/f*^ BMDMs compared to the *tlr4* deletion group by enrichment analysis from TRRUST database. Thus, macrophage TLR4 may not only function as a key regulator for macrophage polarization and activation but may also play a regulatory role in bridging the innate and adaptive immune response by activating MHC-expressing macrophages to present antigens to the T cells and promoting the Th1/Th17-dependent anti-GBM GN.

It should be pointed out that although neutrophils appear to be an early and transient event in the pathogenesis of anti-GBM GN [20, 30], we did find a few NMP-R14^+^ neutrophils during progressive renal injury over days 7 and 14, accounting for about 2% of F4/80^+^ macrophages. However, flow cytometry detected a relatively large CD11b^+^Ly6G^+^ population (9–13%). This discrepancy may be largely associated with the use of different detecting antibodies and methodologies in this study. Indeed, a few NMP-R14^+^ neutrophils were detected in the kidney tissue sections by immunohistochemistry, which is consistent with the previous notion [20, 30]. Whereas, it is highly possible that the relative high numbers of Ly6G^+^ cells by flow cytometry may be associated with the reaction of the anti-Ly6G antibody to some macrophage precursors as previously reported [44, 45]. Thus, although we could not exclude the potential role of neutrophils-derived *tlr4* in the induction phage of anti-GBM GN because *lysM* promoter-driven Cre recombinase also results in functional deficiency of *tlr4* in neutrophils, the massive macrophage infiltration and the majority of macrophage TLR4 deletion (85%) during progressive crescentic GN may reveal a critical role for macrophage-dependent TLR4, rather than neutrophil-derived TLR4, in anti-GBM GN.

In summary, myeloid TLR4 plays a pathogenic role in anti-GBM GN via polarizing M1 macrophage activation while suppressing the M2 phenotype and by shifting the T cell immune response from Th1/Th17 to Treg. Thus, targeting myeloid TLR4 may be a novel therapy for immunologically mediated kidney diseases.

### Supplementary Information

Below is the link to the electronic supplementary material.

Supplementary file1 (PDF 15422 KB)

## Data Availability

All data generated or analysed during this study are included in this published article. The sequencing data are available at the Gene Expression Omnibus website (https://www.ncbi.nlm.nih.gov/geo/) under accession GSE162497. To review GEO accession GSE162497, go to https://www.ncbi.nlm.nih.gov/geo/query/acc.cgi?acc=GSE162497 and enter the secure reviewer token into to box: cvstamiybjojtuz.

## Code availability

Raw counts file for gene expression was uploaded to Rstudio and analyzed by R package named ‘ggplot2’ (version 3.3.2) on CRAN [46]. The source code for this package is available at https://github.com/tidyverse/ggplot2. Network enrichment analysis was performed on Metascape (metascape.org) [31].
