# Supplementary material for: Regulatory role and mechanisms of myeloid TLR4 in anti-GBM glomerulonephritis
Source: Cell Mol Life Sci. 2021 Sep 27;78(19-20):6721–34. doi: 10.1007/s00018-021-03936-1 (PMC8558180; doi:10.1007/s00018-021-03936-1)
Supplement: Supplementary file 1 — Supplementary file1 (PDF 15422 KB) [file 18_2021_3936_MOESM1_ESM.pdf]

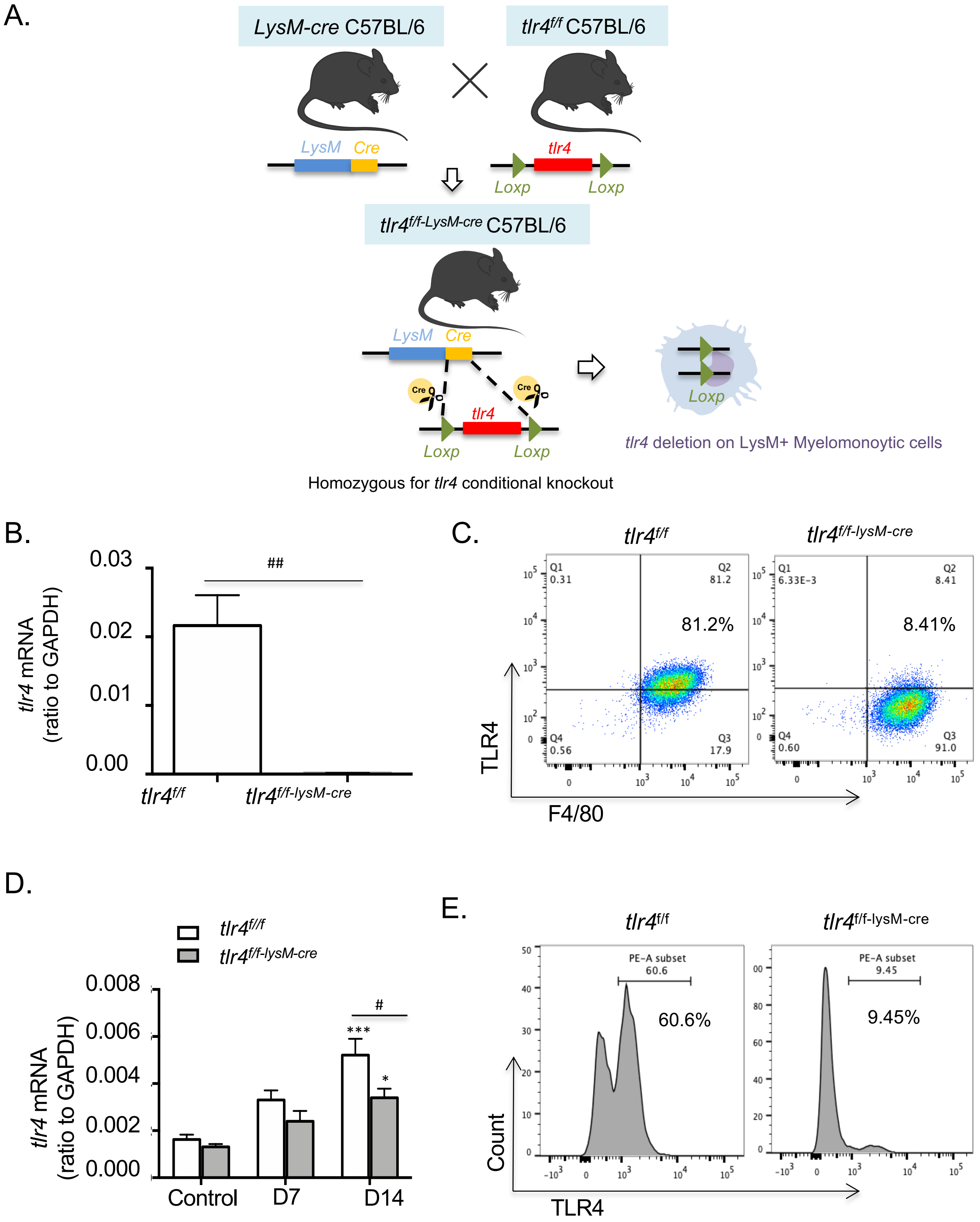

Supplementary Figure 1. Characterization of *tlr4*$^{f/f\text{-}lysM\text{-}cre}$ mice. A. Schematic illustration of mouse breeding to generate the mice with *tlr4* deficiency in macrophages**. B.** Realtime PCR (mean ± SEM, n=3) and **C.** flowcytometric analysis of TLR4 expression on bone marrow derived macrophages (BMDMs) isolated from *tlr4*$^{f/f}$ and *tlr4*$^{f/f\text{-}lysM\text{-}cre}$ mice. **D.** Realtime PCR analysis of *tlr4* mRNA level in renal cortex of control or diseased mice on day 7 and 14 after nephrotoxic nephritis induction (mean ± SEM, n=8); **E.** Representative flowcytometric plots of TLR4 expression on macrophages from the kidney on day 14 after nephrotoxic nephritis induction (gating on live CD45$^+$F4/80$^+$ cells). *p< 0.05, ***p< 0.001 vs. corresponding control; $^{\#}p$< 0.05, $^{\#\#}p$< 0.01 vs. corresponding *tlr4*$^{f/f}$.

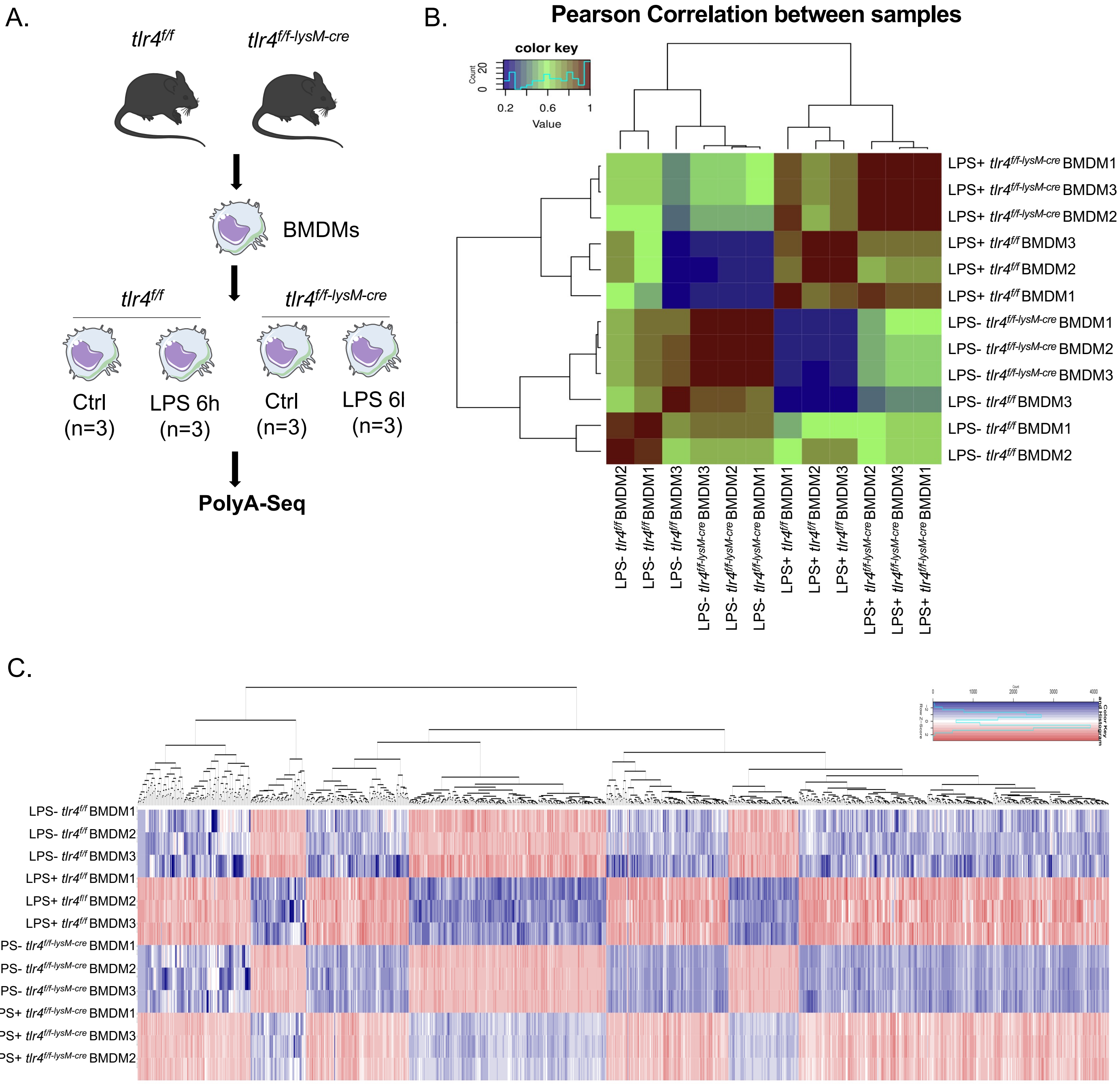

Supplementary Figure 2. PolyA-RNA sequencing analysis of BMDMs isolated from *$tlr4^{f/f}$* and *$tlr4^{f/f\text{-}lysM\text{-}cre}$* mice with or without LPS stimulation. A. Strategy of RNA sequencing of BMDMs isolated from *$tlr4^{f/f}$* and *$tlr4^{f/f\text{-}lysM\text{-}cre}$* mice cultured with or without 1ug/L of LPS for 6 hours; **B.** Heatmap of Pearson correlation analysis showing hierarchical clustering of transcriptome similarity in different groups. Independent replicates of the same cell types or treatments were clearly distinguishable and clustered by overall similarity in their gene expression profiles; **C.** Heatmap of gene expression showing a significant difference between groups.

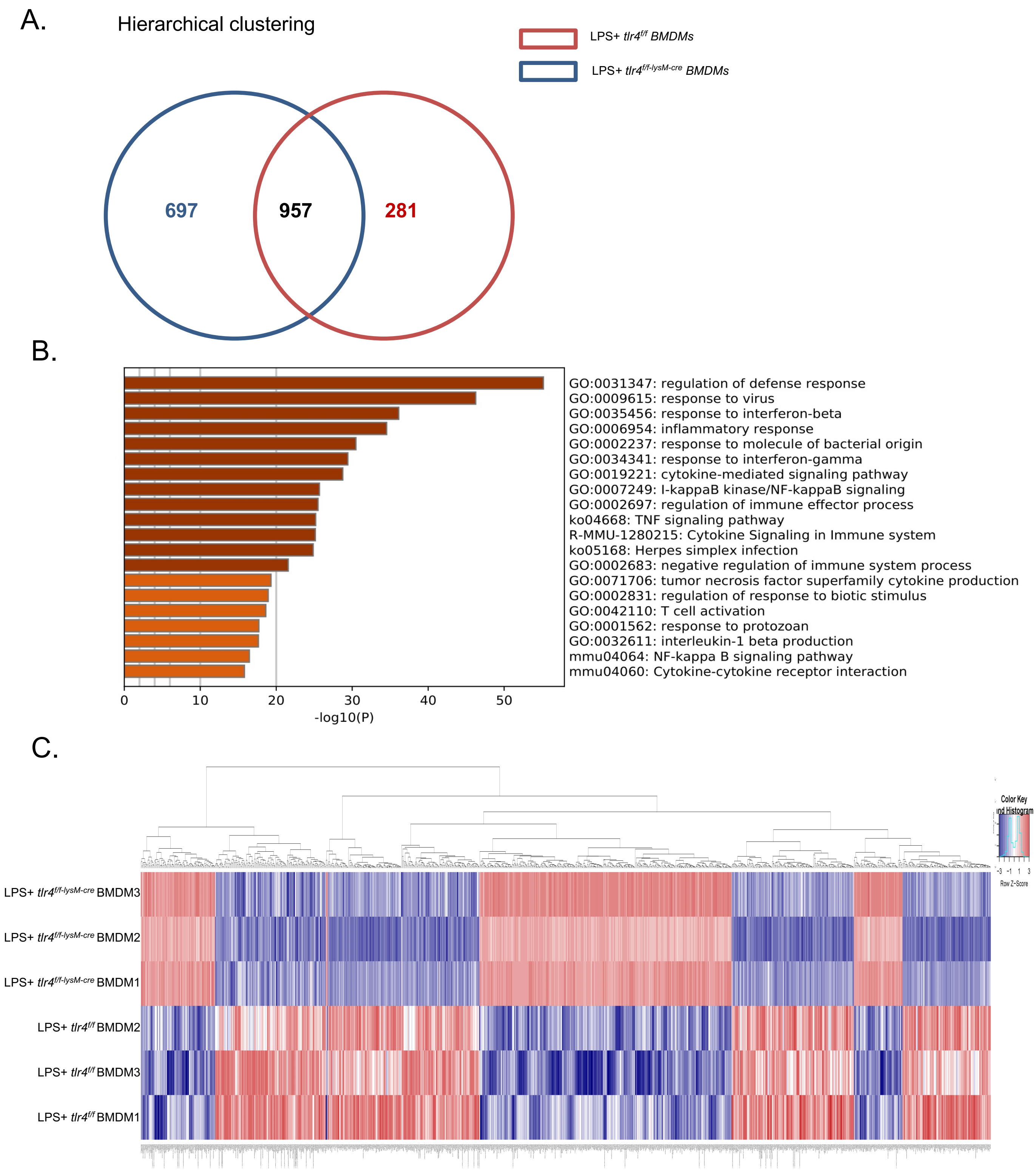

Supplementary Figure 3. PolyA-RNA sequencing analysis captures TLR4-dependent programming of LPS-induced transcriptome profile in BMDMs. A. Venn diagram showing the 697 and 281 unique DEGs found in LPS stimulated *tlr4*$^{f/f\text{-}lysM\text{-}cre}$ and *tlr4*$^{f/f}$ BMDMs, where 957 genes are commonly regulated in both groups. **B.** Heatmap shows top significantly enriched terms across 957 commonly regulated DEGs in LPS treated *tlr4*$^{f/f}$ and *tlr4*$^{f/f\text{-}lysM\text{-}Cre}$ BMDMs, coloured by log10 (FDR); **C.** Heatmap of gene expression showing a significant difference between LPS stimulated *tlr4*$^{f/f\text{-}lysM\text{-}cre}$ and *tlr4*$^{f/f}$ BMDMs.

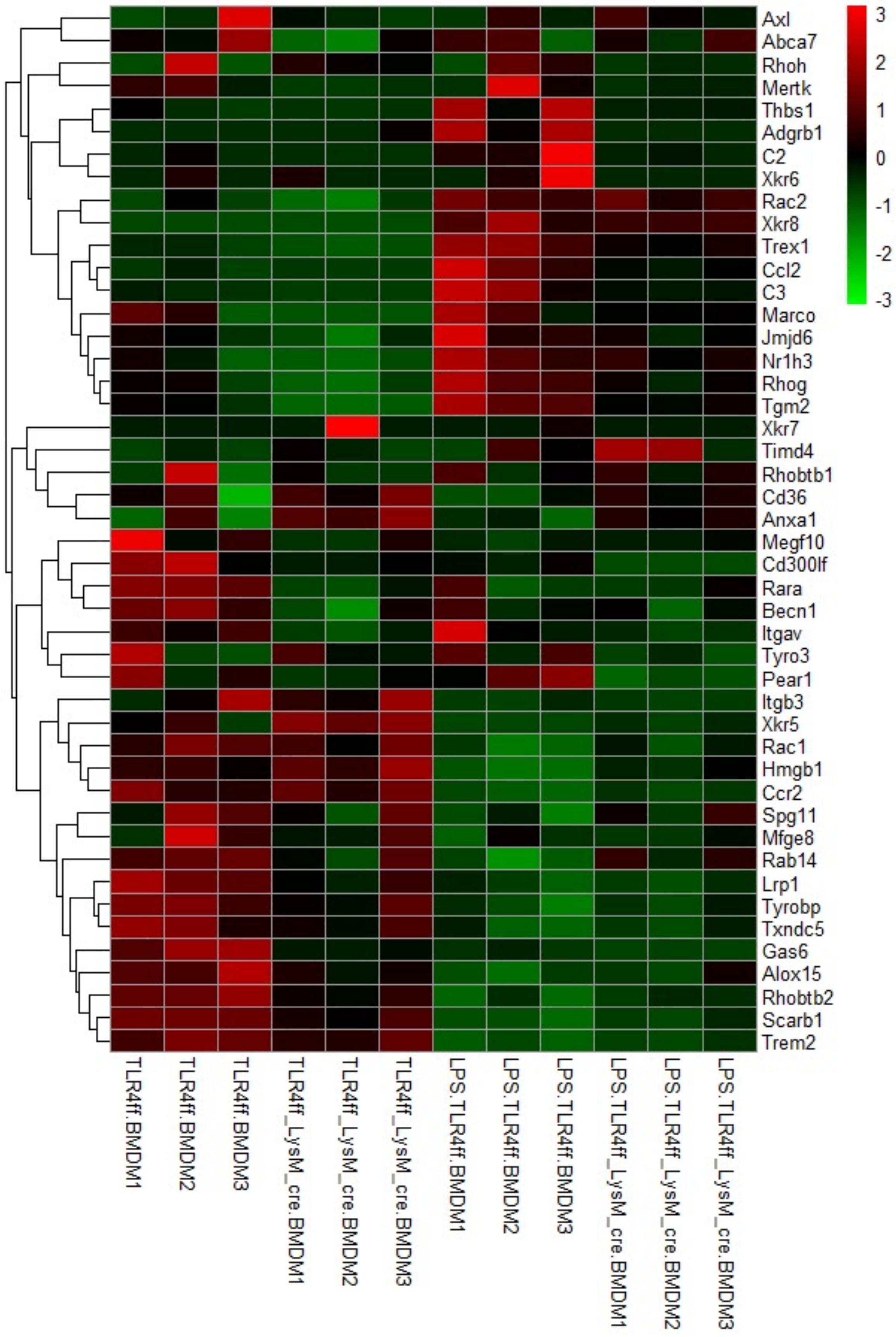

Supplementary Figure 4. Heatmap of efferocytosis-related gene expression in BMDMs. Heatmap showing the relative expression of genes involved in efferocytosis signaling among *tlr4*$^{f/f}$ and *tlr4*$^{f/f\text{-}lysM\text{-}Cre}$ BMDMs with or without LPS stimulation. The complete list of genes is according to GO:0043277 [efferocytosis]. Color indicates the gene expression level, and the average expression (avg. exp) scale is shown on the right.
